# Statin-Induced Necrotizing Autoimmune Myopathy: Case Report of a Patient under Chronic Treatment

**DOI:** 10.1155/2023/6550473

**Published:** 2023-12-16

**Authors:** Ilaria Anna Bellofatto, Marta Sessarego, Amedeo Tirandi, Chiara Olivero, Cosimo Sgura, Elia Maioli, Aurora Gavoci, Elisa Schiavetta, Federica Frè, Benedetta Saccomanno, Federico Zaottini, Riccardo Picasso, Chiara Fiorillo, Luca Liberale, Luciano Carlo Ottonello, Nicholas Bardi, Fabrizio Montecucco

**Affiliations:** ^1^IRCCS Ospedale Policlinico San Martino Genoa-Italian Cardiovascular Network, Larogo Benzi 10, Genoa 16132, Italy; ^2^Radiology Unit, Department of Health Sciences (DISSAL), University of Genoa, Via Pastore 1, Genoa 16132, Italy; ^3^IRCCS Ospedale Policlinico San Martino, Largo Benzi 10, Genoa 16132, Italy; ^4^Pediatric Neurology and Muscular Diseases Unit, University of Genoa and G. Gaslini Institute, Via Gaslini 5, Genoa 16132, Italy; ^5^First Clinic of Internal Medicine, Department of Internal Medicine, University of Genoa, Viale Benedetto XV, No.6, Genoa 16132, Italy

## Abstract

**Introduction:**

3-Hydroxy-3-methylglutaryl coenzyme A reductase (HMGCR) inhibitors are widely used worldwide to treat dyslipidaemia and prevent cardiovascular events. Statins can cause a wide variety of muscle injuries ranging from myalgia to severe rhabdomyolysis. In most cases, these symptoms are mild and self-limiting and do not require specific treatment besides drug withdrawal. Statin-induced autoimmune necrotizing myopathy (SINAM) is a rare but potentially fatal complication, characterized by the subacute onset of progressive proximal muscle weakness and considerably high creatine phosphokinase (CK) levels in patients exposed to statins. The diagnosis is supported by the presence of antibodies HMGCR, which allows the differentiation from other forms of necrotizing autoimmune myopathies. Symptoms usually progress even after statin discontinuation and can determine severe muscle damage. *Summary*. We describe the case of a 77-year-old man who developed SINAM after 5 years of statin use. He suffered from muscle functional impairment mainly involving proximal lower limb muscles which progressed to the point that he almost became bedridden. Initial treatment with prednisone alone was not effective, and he required a combination therapy with steroids, methotrexate, and intravenous immunoglobulins. After 5 months of therapy and rehabilitation, he showed complete laboratory response and muscle strength recovery.

**Conclusion:**

Recognizing SINAM is paramount in order to promptly start treatment and avoid permanent muscle damage. Using a combination therapy from the beginning could contribute to a better outcome. Prompt statin cessation, categorization of the muscle disease by autoantibody testing, imaging, and histology, exclusion of malignancy, and anti-inflammatory therapy with corticosteroids, antimetabolites, immunoglobulins, and in some cases rituximab are currently accepted approaches to this entity.

## 1. Introduction

SINAM has been recently recognized as a distinct clinical entity within the group of adult inflammatory myopathies [1]. It is characterized by the subacute onset of progressive proximal muscle weakness and considerably high CK levels in patients exposed to statins. The presence of antibodies against 3-hydroxy-3-methylglutaryl-CoA reductase (HMGCR) allows the differentiation from other immune-mediated necrotizing myopathies. Anti-HMGCR antibodies have also been observed in a minority of statin-naïve patients, who have similar clinical presentation, but tend to be younger and have higher CK levels than statin-exposed patients [2]. Myopathy can develop several years after statin exposure regardless of the duration of therapy, and it progresses even after statin discontinuation [3]. The estimated incidence of SINAM is two to three out of every 100,000 individuals treated with statins [2]. The most accredited pathogenic mechanism of SINAM involves a break in tolerance caused by up-regulation of HMGCR secondary to statin exposure. Overexpression of HMGCR in regenerating muscle fibres could perpetuate autoimmunity even after statin discontinuation. However, the detection of anti-HMGCR antibodies even in statin-naïve patients suggests a role of genetic and environmental factors [4].

## 2. Case Presentation

**Figure 1:**
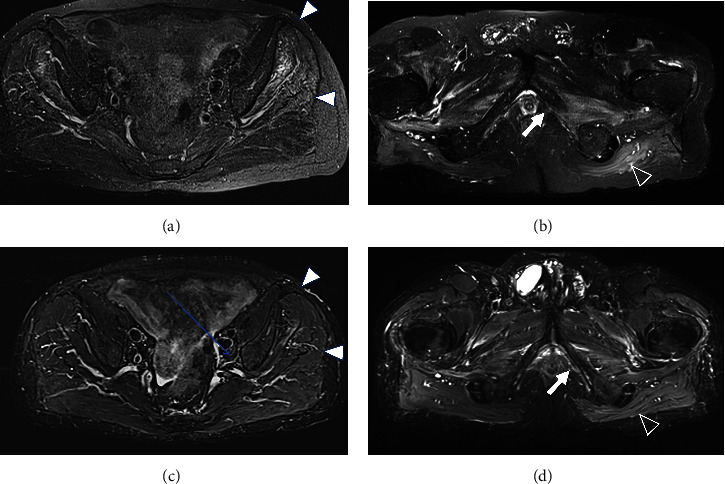
MRI of skeletal muscle. The figure shows four MRI axial views of pelvic girdle muscle acquired before (a, b) and after (c, d) therapy. The images are T2-weighted sequences with chemical-shift fat saturation obtained at the level of greater sciatic foramen (a) and (c) and at the level of ischial tuberosity (b) and (d). In (a), mild hyperintensity of signal within the muscles gluteus minimun and medium (arrowhead) on the left side can be observed, and it is related to intramuscular edema. After therapy, the edema is significantly reduced (arrowhead in (c)). Similarly, in the more caudal scan, the edema related to the inflammatory process can be appreciated in the left obturator externus, adductor magnus muscles (arrow in (b)) and left gluteus maximum muscle (void arrowhead in (b)). After therapy, the intramuscular edema of obturator externus and adductor magnus is less evident (arrow in (d)) while it is completely resolved in the gluteus maximus (void arrowhead in (d)). The muscle pathologic signal alterations are bilateral but on the right side they result more faint, keeping with the patchy and asymmetrical muscular involvement of the statin-induce myonecrosis.

**Figure 2:**
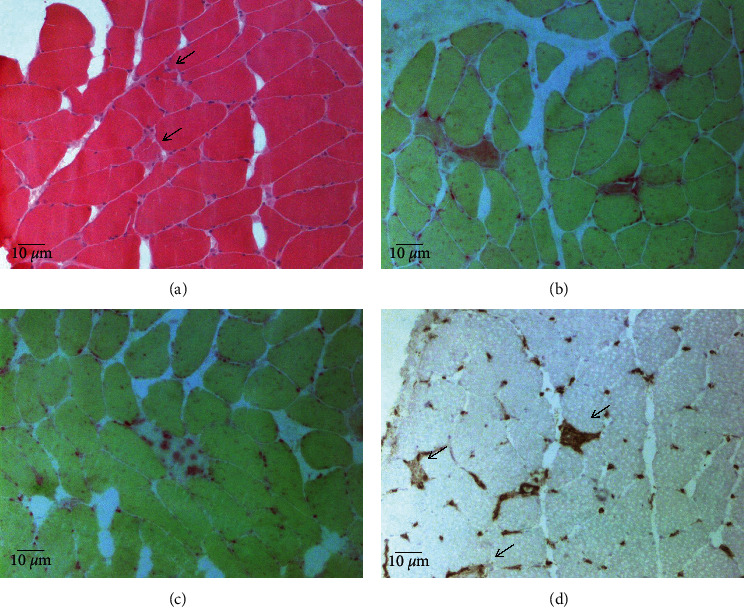
Histology of skeletal muscle biopsy. (a) Haematoxylin-Eosin (HE) staining shows variation in fibres size and morphology, increased endomyisial connective tissue and several degenerating fibres (arrows). Magnification 20x. (b) Acid phosphatase staining reveals increased activity in the necrotic-degenerating fibres scattered among different fascicles. Magnification 20x. (c) Acid phosphatase staining marks intensively a necrotic fibre with macrophages infiltration. Magnification 20x. (d) Immunoperoxidase reaction with antibody against HLA1/MHC1 complex is present on the necrotic/degenerating fibres. Magnification 20x.

**Figure 3:**
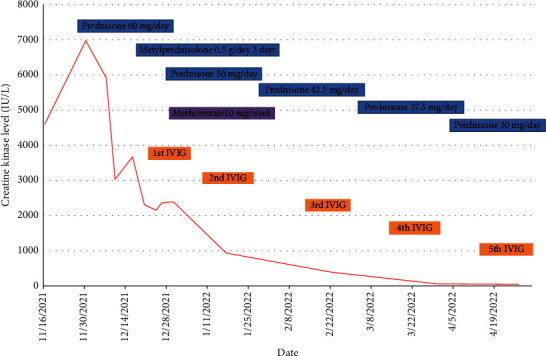
Creatine kinase (CK) levels and response to therapy over time.

A 77-year-old man presented to our attention complaining of weakness and myalgia of the lower limbs for about a month. His past medical history revealed ischemic heart disease in 2017 (when he was started on secondary prevention statin therapy), thyroidectomy, localised colorectal cancer resection 10 years before, benign prostate hyperplasia, and abdominal aorta aneurysm. At the time of admission, medications include atorvastatin 40 mg/day, aspirin 100 mg/day, ramipril 2.5 mg/day, levothyroxine 50 *μ*g/day and 75 *μ*g/day on alternate days, alfuzosin 10 mg/day, and dutasteride 0.5 mg/day. On examination, the patient showed good muscle tone according to his age in both upper and lower limbs. Lower limb strength was graded 4/5 on the Medical Research Council (MRC) scale at the gluteus muscles and the iliopsoas. At that point, there was neither power loss of muscle strength in the upper limbs nor clear gait impairment while patient referred to easy exhaustion when walking. Blood tests showed a considerable increase in creatine phosphokinase (CK), lactate dehydrogenase (LDH), and alanine aminotransferase (ALT) levels which were 4598 IU/L, 695 IU/L, and 401 IU/L, respectively. Whereas full blood count, inflammatory markers, coagulation tests, renal function, urea, electrolytes, and liver function were normal. Thyroid function tests unveiled a poorly controlled hypothyroidism with TSH level of 11.49 mIU/L (n.v. 0.270–4.200 mIU/L) and T4 level of 8.02 ng/L (n.v. 9.30–17 ng/L); therefore, levothyroxine dose was increased up to 75 *μ*g per day. Atorvastatin was immediately suspended. Autoimmunity tests reported the presence of anti-nucleus antibodies (ANA) with a titre of 1 : 160 and mixed speckled/nucleolar pattern. Myositis antibody panel was negative (Table 1). In order to rule out malignancy in the context of a paraneoplastic syndrome, we performed a CT scan of the chest and abdomen, which proved negative. Considering the clinical stability of the patient and the flattened CK levels, after 10 days, the patient was discharged with the plan of undergoing an MRI scan of the lower limbs and a muscle biopsy. Also, serum samples were sent to the closest facility performing an anti-HMGCR antibodies assay, and the results were pending. After one week, the patient was hospitalized again due to worsening symptoms. Lower limb weakness slightly progressed (gluteus muscles strength: 3/5; iliopsoas strength: 4/5) while waddling gait was evident. He could barely stand up without help, and to get a sitting position from lying on the bed, he required the use of alternative, distal muscles. Furthermore, he complained of mild upper-limb functional impairment and dysphagia with solid food. CK levels markedly increased up to 6978 IU/L. An MRI scan of the lower limbs showed bilateral intramuscular oedema with patchy distribution involving the gluteus minimus and medius muscles, the insertional area of gluteus maximum, iliopsoas, the adductor magnus, adductor longus, obturator externus, obturator internus muscles, and the central aponeurosis of rectus femoris muscles (Figure 1). There was an initial fatty replacement involving the gluteus muscles and the adductor magnus. The alterations were more evident along the intramuscular aponeurosis and myotendinous junctions. Electromyography showed myopathic changes, whereas muscle biopsy highlighted the presence of necrosis with macrophages infiltration and thickening of connective tissue with mild inflammatory changes (Figure 2). Anti-HMGCR antibody titre was 299.7 CU/ml (n.v. <20 CU/mL). Based on these results, a diagnosis of statin-induced autoimmune necrotizing myopathy (SINAM) was made, and the patient was prescribed with prednisone 1 mg/kg/day (60 mg/day). In the following 5 days, CK levels dropped to 3032 IU/L in stark contrast with the clinical picture that only minimally improved. On the 13th day of steroid treatment, we witnessed a further clinical deterioration. The patient was no longer able to lift both legs against gravity and could only walk with assistance. Despite the overall stability of CK levels, intravenous immunoglobulin therapy was started at the dose of 0.4 g/kg per day for 5 days. At the end of the cycle, we challenged the patient with a three-day course of 0.5 g of methylprednisolone before returning to the previous dosage of prednisone. After a few days, methotrexate was added at the dosage of 10 mg per week. In light of a slow but persistent clinical and laboratory improvement, we began a careful tapering of prednisone with the plan of reducing the dosage by approximately 5 mg each month. An important aspect to highlight is the challenge we found in creating a rehabilitation plan for this patient, given the lack of clear evidence. During the acute phase of disease, we opted for a resting strategy limiting physical activity to preserve muscle tissue from further damage. In the second stage, during disease remission, our patient started a gradual rehabilitation process with progressive active physiotherapy. The patient was discharged after a second cycle of immunoglobulins with CK levels of 880 IU/L. Since then, we are following the patient in our outpatient unit. He recently completed the 6^th^ monthly infusion of immunoglobulins out of the twelve planned. CK levels completely normalised and upper limb function has fully recovered (Figure 3). Minimal weakness of the proximal lower limbs still remains without affecting the patient's gait. A three-year follow-up for cancer screening has been scheduled and will be recommended.

## 3. Discussion

There is currently no consensus on diagnostic criteria for SINAM, and diagnosis is based on a combination of clinical, laboratory, and pathologic features. Moreover, the exclusion of other myopathies with similar characteristics is paramount. Patients typically present with subacute symmetrical muscle weakness, mainly affecting the proximal regions of the upper and lower limbs, with or without myalgia. Lower limbs are predominantly involved, but neck muscle weakness and dysphagia might be present [5]. Bulbar muscle involvement has also been described as having a high mortality rate despite aggressive treatment. Conversely, extra-muscular involvement is rare as opposed to other autoimmune myopathies. CK levels are markedly and persistently raised to the order of several thousand, whereas inflammatory markers are negative. Electromyography shows nonspecific elements of active myopathy, whereas the MRI scan highlights muscle oedema associated with inflammation or myofiber necrosis and fatty replacement in cases of irreversible muscle damage. Distinctive features of muscle-biopsy specimens from patients with SINAM include muscle cell necrosis and macrophages infiltration. Only a small percentage of biopsy specimens presents a lymphocytic inflammatory exudate which is a pathologic hallmark of other immune-mediated myopathies. Upregulation of major histocompatibility complex class I molecules is also frequent [2]. The detection of anti-HMGCR antibodies in statin-exposed patients who develop myopathy is highly specific and strongly supports the diagnosis [2]. Whether they have a direct pathological action on muscle fibres or not is controversial and requires further studies. However, there is a good correlation between HMGCR antibodies, CK levels, and strength in patients with SINAM which makes them a good marker of disease activity. As a consequence, serial determinations of HMGCR antibodies during the disease course might be useful to monitor response to treatment, although in several instances antibody titre never returns to normal even in patients after full recovery.

Another important aspect is the link between SINAM and cancer. The association of autoimmune myopathies with cancer is well documented, especially for dermatomyositis [6]. The term “cancer-associated myositis” is commonly used to define a malignancy manifesting three years before or after the diagnosis of myositis [7]. Patients with anti-HMGCR antibodies, particularly those aged 50 or above, also demonstrate an increased probability of developing cancer with the highest risk observed within the first year of myopathy diagnosis [8]. Consequently, cancer screening should be continued at least annually for three years after the detection of anti-HMGCR antibodies.

Treatment of SINAM is largely based on expert consensus and clinical case series; as to date, no randomized controlled trials have been conducted. The evidence show that the best induction strategy consists of a combination of steroid therapy and immunosuppressants such as methotrexate, azathioprine, or mycophenolate mofetil in combination with intravenous immunoglobulins [9, 10]. Also, the use of rituximab is becoming gradually more common both to induce remission and to prevent relapses in refractory patients [2]. Monotherapy with glucocorticoids might not be efficacious, as it was in our experience, albeit oral prednisone can be a reasonable first approach in patients with mild weakness [1, 11]. Likewise, the use of intravenous immunoglobulins alone has been described and might be an option for selected individuals [12]. The exact duration of treatment after remission is unknown as there is significant individual variability in clinical response. Many patients relapse during tapering or withdrawal of therapy, hence the importance of close monitoring [12]. Furthermore, in some cases, muscle weakness persists despite normalisation of CK levels due to permanent damage from fatty replacement of muscle tissue. Lastly, it is important to treat possible concomitant conditions favouring myopathy development. Here, we increased the dosage of levothyroxine as hypothyroidism is a known risk factor for muscle damage [13].

## 4. Conclusions

SINAM is a rare but potentially life-threatening disease which can appear with a variable latency after statin use. Early diagnosis is paramount in order to avoid extensive muscle damage and increase the chances of the good strength recovery. Symptoms can progress despite withdrawal of statins, and aggressive therapy with a combination of steroids, immunosuppressants, and intravenous immunoglobulin is necessary in most cases. However, an individualised approach is needed as relapses with therapy withdrawal or tapering are frequent. We observed a mismatch between laboratory and clinical response to therapy, with a slower muscle strength recovery in contrast to a rapid CK level decline. With regards to rehabilitation timing, we did not find significant data in the literature to guide our practice. Based on our experience, it appears reasonable to wait until a sustained clinical and laboratory response before starting active physiotherapy, in order not to add further damage to an already stressed muscle.

## Figures and Tables

**Table 1:** Myositis antibody panel.

Mi-2 Alpha
Mi-2 Beta
TIF-1 Gamma
MDA5
NXP2
SAE1
KU
PM-Scl 100
PM-Scl 75
Jo-1
SRP
PL-12
EJ
0J
PL-7
